# Redefining communities: The association between deferred action, online and offline social capital and depressive symptoms among undocumented young adults

**DOI:** 10.1016/j.pmedr.2021.101563

**Published:** 2021-09-20

**Authors:** May Sudhinaraset, Amanda Landrian, Hye Young Choi, Irving Ling

**Affiliations:** a Community Health Sciences, Fielding School of Public Health, University of California, Los Angeles, 650 Charles E. Young Dr. South, Los Angeles, CA 90095, USA; b Social and Behavioral Sciences, Harvard T.H. Chan School of Public Health, 677 Huntington Avenue, Boston, MA 02115, USA; c Department of Medicine, University of California, San Francisco, 505 Parnassus Ave., San Francisco, CA 94143, USA

**Keywords:** Undocumented immigration, Young adults, Social capital, Online social capital, Mental health, Deferred action for childhood arrivals

## Abstract

An explosion in Internet use, social networking sites, and COVID-19 has promoted a new concept in health – online social capital, defined as linkages to online social networks that promote trust and group norms. Particularly for the 1.3 million undocumented young adult immigrants who “live in the shadows,” the Internet may serve as a place of support and information. This study examines the association between documentation status (defined as Deferred Action for Childhood Arrivals (DACA) status), offline social capital, online social capital, and depressive symptoms among foreign-born Latino and Asian and Pacific Islander young adults in California (N = 208) using data from an internet-based survey conducted in 2017. This study found that those without DACA status had higher online social capital (p < 0.001) and increased depressive symptoms (p = 0.01) than those with DACA status. Using linear regression, we found evidence of online social capital potentially mediating the relationship between DACA status and depressive symptoms. This study also found that as offline social capital increases, the association between online social capital on depressive symptoms decreases. This study points to the power of offline communities and the importance of increasing access to community resources, particularly to those without documentation status who may only have online social networks.

## Highlights

• This study assesses DACA status, offline and online social capital on depressive symptoms among undocumented young adults.
• DACA-recipients have higher levels of offline social capital compared to non-recipients.
• DACA-recipients have lower depressive symptoms compared to non-recipients.
• Online social capital partially mediates DACA status and depressive symptoms.
• As offline social capital increases, the effect of online social capital on depressive symptoms decreases.

## 1 Introduction

The Internet has seen an explosion of users, redefining “community” as online spaces that have infiltrated the lives of young people globally. Popular online social networking sites like Facebook, for example, had over 2.4 billion users worldwide in 2019 (Facebook users Worldwide, 2019). Movements such as Occupy Wall Street, the Arab Spring, and Black Lives Matter demonstrate the role that social media plays in collective action and galvanizing and leveraging offline social movements and resources (Ince et al., 2017, The Role of Social Media in the Arab Uprisings|Pew Research Center [Internet], 2012, Wolfsfeld et al., 2013). A high proportion of immigrants use the Internet, with almost 80% of foreign-born individuals in California having broadband Internet connectivity at home (Annual Survey, 2017). In many ways, and particularly during the COVID-19 pandemic, the Internet is redefining the concept of “community” – one that lacks borders and physical spaces and instead is built on the concept of connection and sense of belonging. This rise in Internet use and social networking sites has promoted a new concept in health – online social capital, defined as linkages to online social networks that promote trust and group norms (Williams, 2006). These online social networks may develop via forums, social media platforms, phone and video communication services, online games, and other online communication platforms (Spottswood and Wohn, 2020).

Particularly for undocumented immigrants who “live in the shadows,” the Internet may serve as a sanctuary space and place of support and information. Indeed, this is seen with regards to applications and websites such as *Tarjimly, Arrived*, and *Notifica* which provide news about immigration raids, safe routes of travel, and safe houses. Among Syrian refugees fleeing to Europe, *WhatsApp* (free texting/calling application over the Internet) has been used to pass along information and warnings to others making similar journeys (Manjoo, 2016).

There are currently 10.7 million undocumented immigrants, of which, 1.3 million are estimated to be young adults eligible for Deferred Action for Childhood Arrivals (DACA) (Zong et al., 2019), a federal program enacted in 2012 that grants temporary deportation relief and work authorization for eligible applicants. DACA continues to be in limbo, as a federal judge blocked new applications in July 2021 (Sacchetti, 2021). However, DACA is associated with improved mental health status (Gonzales Bautista-Chavez, 2014, Patler and Laster Pirtle, 2018, Venkataramani et al., 2017), potentially through increased social ties (Sudhinaraset et al., 2017). Undocumented young adults experience sustained stress due to fear of deportation, family separation, and experiences of exclusion (Gonzales et al., 2013). Research also finds that lack of legal status is associated with psychological distress (Patler and Laster Pirtle, 2018) and increased risks of anxiety and depression (Potochnick and Perreira, 2010, Garcini et al., 2017).

To our knowledge, no study has assessed the role of online social capital among undocumented young adults, despite considerable engagement in this new space, and concerns regarding surveillance, privacy, and harassment by anti-immigrant groups (Guberek et al., 2018). Moreover, few studies on mental health among undocumented young adults directly assess documentation status, instead relying on imperfect proxy measures (Ro and Van Hook, 2021).

### 1.1 Research questions

The objective of this study was to examine the associations between DACA status, offline social capital, online social capital, and depressive symptoms among undocumented Latino and API immigrant young adults. Specifically, we address the questions: 1) How does DACA status influence online social capital, offline social capital, and depressive symptoms?; 2) Does online social capital mediate the relationship between DACA status and depressive symptoms?; 3) Does offline social capital mediate the relationship between DACA status and depressive symptoms?; and 4) What is the extent to which offline social capital moderates the relationship between DACA status, online social capital and depressive symptoms?

### 1.2 Conceptual framework

**Fig. 1:**
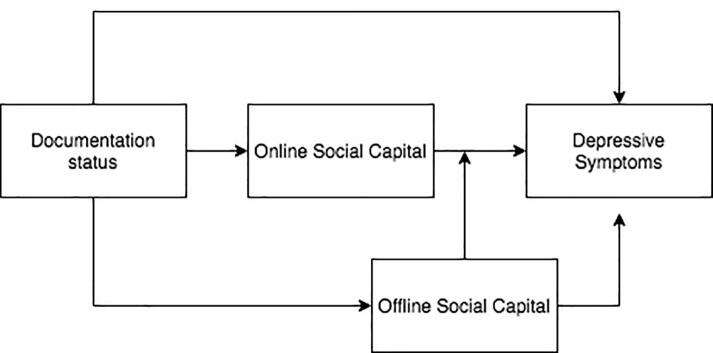
Model of DACA Status, Online Social Capital, Offline Social Capital, and Depressive Symptoms.

To provide a simple conceptual framework, we adapted the Stress Process Model to illustrate the influence of documentation status on depressive symptoms (Fig. 1) (Pearlin et al., 1981, Turner, 2009). First, we posit that DACA both directly and indirectly decreases depressive symptoms (Patler and Laster Pirtle, 2018) through offline social capital by increasing social networks through work opportunities, education, and community organizations (Sudhinaraset et al., 2017). Second, we hypothesize that online social capital may be a marker of immigrant-related stress and mediates the relationship between documentation status and depressive symptoms. That is, while undocumented young adults may find refuge and information on the Internet, online social capital may reflect increased stress, lack of offline social networks, and inability to connect to physical spaces.

The framework also posits that social resources moderate, or buffer, the relationship between stress and mental health. There is strong evidence for an inverse relationship between social capital and common mental disorders (Ehsan and De Silva, 2015). However, it is unclear how relying on social networks online affects psychological wellbeing. The time displacement model hypothesizes that Internet use would be associated with social isolation and depression, with the belief that time spent online erodes the ability to cultivate in-person relationships (Nie and Erbring, 2002, Nie and Hillygus, 2002). In the conceptual framework, we posit that offline social capital moderates the relationship between online social capital and psychological wellbeing. That is, while high online social capital may be a marker of immigrant-related stress, high levels of offline social capital may buffer that association.

## 2 Methods

### 2.1 Procedures and participants

This study utilizes data from the Building community Raising All immigrant Voices for health Equity (BRAVE) Study, an internet-based survey conducted from June 2017 to August 2017 among undocumented Latino and Asian and Pacific Islander (API) immigrant young adults living in California. The BRAVE Study engaged with a community advisory board (CAB) comprised of experts from public health and health policy, education, and immigration advocacy, as well as individuals from the undocumented community who helped with survey development, participant recruitment, and data interpretation. Eligibility included those who were between the ages of 18 and 31 years, identified as undocumented, Latino or API, and currently residing in California, and were able to take the survey in English. Because there is no sampling frame for undocumented young adults, participants were recruited using multiple strategies, including audience-targeted Facebook advertisements, organization email listservs, and snowball sampling. Steps were taken to ensure data quality and validity, including methods preventing participants from taking the survey multiple times. The Institutional Review Board at the University of California, San Francisco approved this study and all participants provided informed consent.

### 2.2 Dependent variable

The dependent variable was depressive symptoms as measured using the 10-item Center for Epidemiologic Studies Depression Scale Revised (CESD-R) (Cronbach α = 0.86) (Björgvinsson et al., 2013), which has been used among other young adult and immigrant populations (Davila et al., 2014, Stein et al., 2014, Ro et al., 2021). Items are scored from “0″ (rarely or none of the time) to 3 (“all of the time”) to assess the frequency of depressive symptoms. Total scores range from 0 to 30 with higher scores indicating greater severity of depressive symptoms.

### 2.3 Independent variables

DACA status was the primary independent variable of interest and assessed with a direct question: “Are you currently a DACA recipient” (1 = Yes, 0 = No). Online social capital and offline social capital were included as a potential mediator and moderator, respectively, and were measured using a subset of items from the Internet Social Capital Scales (ISCS) (Williams, 2006). The original scale consisted of 20 items; however, we chose a subset of nine items based on the need to keep our survey brief. The subset of items was chosen to focus on emotional social capital or being connected to a larger community as opposed to financial social capital. For example, we did not use questions that focused on being provided loans or job references. Questions asked participants to indicate the extent to which they agreed with various statements (e.g., “there are several people online/offline I trust to help solve my problems”) on a 5-point Likert scale ranging from strongly agree to strongly disagree (see Appendix A). Total scores for both online and offline social capital range from 0 to 36 with higher scores indicating greater social capital along that domain (Cronbach α for online and offline social capital items, respectively, were 0.82 and 0.80). Sociodemographic characteristics included race and ethnicity (Latino or API), highest level of education, whether the participant is currently in school, age, gender, employment status, and years in the US.

### 2.4 Analyses

A total of 456 participants completed the survey, of which 427 (94%) recorded responses indicating DACA status. Based on validation of participants using logic edits (see Results) and completeness of data, the final analytic sample included 208 participants. All analyses were conducted in StataSE version 15.1. Chi-square tests (for categorical variables) and two-sided independent sample t-tests (for continuous variables) were used to analyze differences in characteristics by DACA status. Linear regression analyses were used to test the hypothesized pathways, outlined in Fig. 1, as guided by Baron and Kenny’s four step approach for establishing mediation (Baron and Kenny, 1986). First, a simple linear regression was used to confirm a significant relationship between the independent variable, DACA status, and the dependent variable, depressive symptoms (Model 1). Next, a simple linear regression was used to assess the relationship between the independent variable, DACA status, and each of the potential mediators under study – online social capital (Model 4) and offline social capital (Model 5). Finally, multiple linear regression was used to examine whether each of the mediators – online social capital (Model 2) and offline social capital (Model 3) – are significantly associated with the focal dependent variable, depressive symptoms, when DACA status is controlled. Models 2 and 3 were also examined to assess the change in the independent variable, DACA status, when social capital is controlled, comparing the significance and magnitude of the variable coefficients as found in Model 1 to that in Models 2 and 3. According to Baron and Kenny, complete mediation is present when the independent variable is statistically nonsignificant when the mediator is controlled, and partial mediation is present when the independent variable remains statistically significant but the coefficient reduces in magnitude. Following these procedures, multiple linear regression was used to assess: the relationship between DACA status and online/offline social capital and depressive symptoms after controlling for sociodemographic characteristics (Model 6); and the role of offline social capital as a potential moderator of the relationship between online social capital and depressive symptoms (Model 7) after controlling for sociodemographic characteristics.

## 3 Results

Of the 427 participants who recorded responses indicating their DACA status, 219 (51%) were excluded from analyses following logic edits using DACA eligibility requirements (n = 209) or because they did not have complete data for CESD-R items (n = 10). Specifically, participants who endorsed being DACA-recipients but who also indicated being 31 years of age or older as of June 15, 2012, having a previous criminal felony conviction, entering the US after the age of 16 years, and/or having lived in the US for<5 years at the time of the survey were excluded. As a result, the final analytic sample included the remaining 208 participants with complete and valid data on DACA status and CESD-R scores.

Sociodemographic characteristics among the sample and stratified by DACA status are provided in Table 1. Significant differences in race/ethnicity, gender, education, current schooling status, and years in the US were detected by DACA status. Briefly, a higher proportion of those with DACA status identified as Latino whereas a higher proportion without DACA identified as API. Those with DACA status were also more likely to be female, have a college education or higher, and to have lived in the US for at least 11 years than those without DACA status. No differences in current employment were detected across DACA status.

**Table 1:** Sociodemographic characteristics among the total sample and stratified by DACA status.

Characteristic	Total (N = 208)	Stratified by DACA Status		p-value
		DACA (N = 75)	No DACA (N = 133)	
**Race/Ethnicity, n (%)**				<0.001
Latino	102 (49.0)	62 (82.7)	40 (30.1)	
API	106 (51.0)	13 (17.3)	93 (69.9)	
**Age (years), mean (SD)**	23.17 (3.05)	22.68 (3.32)	23.44 (2.86)	0.08
**Gender, n (%)**				<0.001
Male	129 (62.0)	27 (36.0)	102 (76.7)	
Female	79 (38.0)	48 (64.0)	31 (23.3)	
**Education, n (%)**				<0.001
High school or less	47 (22.6)	13 (17.3)	34 (25.6)	
Some college	126 (60.6)	38 (50.7)	88 (66.2)	
College or higher	35 (16.8)	24 (32.0)	11 (8.3)	
**Currently in School, n (%)**				<0.001
Yes	110 (52.9)	53 (39.9)	57 (76.0)	
No	98 (47.1)	80 (60.2)	18 (24.0)	
**Currently Employed, n (%)**				0.86
Yes	143 (68.8)	51 (68.0)	92 (69.2)	
No	65 (31.3)	24 (32.0)	41 (30.8)	
**Years in the US, n (%)**				<0.001
5 years or less	34 (16.4)	5 (6.7)	29 (21.8)	
6 to 10 years	97 (46.6)	7 (9.3)	90 (67.7)	
11 or more years	77 (37.0)	63 (84.0)	14 (10.5)	

*Notes*: DACA = Deferred Action for Childhood Arrivals. API = Asian or Pacific Islander. Chi-square tests were used to assess differences by categorical variables and DACA status. A two-sided independent sample *t*-test was used to assess differences in mean age by DACA status.

Table 2 provides summary statistics for online social capital, offline social capital, and CESD-R scores among the total sample and stratified by DACA status. Significant differences in online social capital scores were detected by DACA status, whereby those without DACA status had significantly higher mean scores (i.e., greater online social capital) than those with DACA status (24 vs. 19, respectively; p-value < 0.001). No significant differences in offline social capital scores were detected. The mean CESD-R score was significantly higher among those without DACA status than those with DACA status (14 vs. 12, respectively; p-value = 0.01). Nearly three-quarters (74%) of the total sample met the clinical cutoff for depression, with a significantly higher proportion of those without DACA status meeting the clinical cutoff than those with DACA status (82% vs. 60%, respectively; p-value = 0.001) (data not shown).

**Table 2:** Mean total online social capital, offline social capital, and CESD-R scores among the total sample and stratified by DACA status.

Characteristic	Total (N = 208)	Stratified by DACA Status		
		DACA (N = 75)	No DACA (N = 133)	p-value
Online social capital score, mean (SD; range)	22.2 (6.6; 0–36)	18.9 (8.4; 0–36)	24.2 (4.3;10–34)	<0.001
Offline social capital score, mean (SD; range)	23.5 (5.5; 0–36)	24.1 (8.0; 0–36)	23.2 (3.4; 9–32)	0.25
CESD-R score, mean (SD; range)	13.6 (5.3; 2–29)	12.4 (6.3; 2–27)	14.3 (4.6; 5–29)	0.01

Notes: DACA = Deferred Action for Childhood Arrivals. CESD-R = Center for Epidemiologic Studies Depression Scale Revised. SD = Standard deviation. Two-sided independent sample t-tests were used to assess differences in mean scores by DACA status.

Results of the various linear regression models used to test for mediation are presented in Table 3. Those with DACA status have an average CESD-R score that is 1.88 points lower than those without DACA status (95% CI: −3.37, −0.38; p < 0.05) (Model 1). When adding online social capital to the model (Model 2), the average CESD-R score increased by 0.17 points for each 1-point increase in online social capital score (95% CI: 0.06, 0.29; p < 0.01) (Model 2). Similarly, we find that offline social capital is significantly associated with depressive symptoms, whereby each 1-point increase in offline social capital score is associated with a decrease of 0.14 points in CESD-R score (95% CI: −0.27, −0.01; p < 0.05). Comparing the coefficient of DACA status when controlling for social capital to that found in Model 1, we find that the variable is no longer statistically significant in Model 2 (indicative of potential complete mediation) and has decreased in magnitude, although remained statistically significant in Model 3 (indicative of potential partial mediation). Finally, in Models 4 and 5, which assess the relationship between DACA status and online and offline social capital, respectively, we find that DACA status is significantly associated with online social capital but not offline social capital. Specifically, those with DACA status have an average online social capital score that is more than 5 points lower than those without DACA (coefficient = -5.30, 95% CI: −7.05, −3.56; p < 0.001). Because DACA status is not significantly associated with offline social capital, it fails to meet all criterion necessary for establishing mediation.

**Table 3:** Testing for mediation: Linear regressions of depressive symptoms on DACA status and social capital.

Predictor	CESD-R Score			Online Social Support	Offline Social Support
	Model 1	Model 2	Model 3	Model 4	Model 5
Constant	14.28 (13.38, 15.18)***	10.12 (7.19, 13.05)***	17.60 (14.46, 20.74)***	24.16 (23.11, 25.20)***	23.17 (22.23, 24.11)***
DACA status					
No	Ref	Ref	Ref	Ref	Ref
Yes	−1.88 (-3.37, −0.38)*	−0.97 (-2.56, 0.62)	−1.75 (-3.23, −0.26)*	−5.30 (-7.05, −3.56)***	0.91 (-0.66, 2.47)
Online social capital		0.17 (0.06, 0.29)**			
Offline social capital			−0.14 (-0.27, −0.01)*		

Adjusted R^2^	0.0242	0.0589	0.0416	0.1449	0.0015

*Notes*: Coefficient (95% confidence intervals) are shown. CESD-R = Center for Epidemiologic Studies Depression Scale Revised. DACA = Deferred Action for Childhood Arrivals. Ref = Referent category. Model 1 tests for significant relationship between DACA status and depressive symptoms. Models 2 and 3 test for a significant relationship between online social capital and offline social capital, respectively, and depressive symptoms when controlling for DACA status. Models 4 and 5 test for a significant relationship between DACA status and online social capital and offline social capital, respectively.

*p < 0.05, **p < 0.01, ***p < 0.001.

**Fig. 2:**
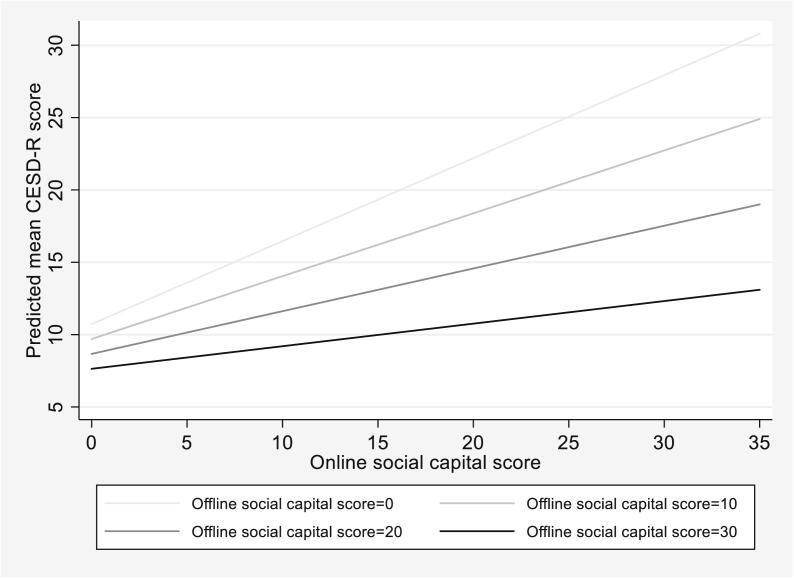
Effect of online social capital on predicted mean CESD-R score as a function of offline social capital score.

In Model 6, assuming all else equal, we find that DACA status, online social capital, and offline social capital are all statistically significantly associated with CESD-R score (Table 4). In short, those with DACA status have a statistically significantly lower average CESD-R scores than those without. Further, following the same trends as found in Models 2 and 3 when only controlling for DACA status, we find that online social capital remains positively associated while offline social capital remains negatively associated with CESD-R score, respectively, when additional demographic characteristics are controlled. Finally, the significance of the interaction term between online social capital and offline social capital added in Model 7 indicates that the association between online social capital and mean CESD-R score differs by offline social capital score. This is further illustrated in Fig. 2, which depicts the relationship between online social capital score and mean predicted CESD-R score as a function of offline social capital scores (i.e., at offline social capital scores equal to 0, 10, 20, and 30). The figure shows that, as offline social capital increases, the association between online social capital on depressive symptoms decreases.

**Table 4:** Multiple linear regression of depressive symptoms on DACA status and social capital.

Predictor	CESD-R Score	
	Model 6	Model 7
Constant	19.52 (12.78, 26.78)***	15.28 (8.06, 22.49)***
DACA status		
No	Ref	Ref
Yes	−3.01 (-5.00, −1.02)**	−2.80 (-4.76, −0.84)**
Online social capital	0.27 (0.15, 0.38)***	0.57 (0.34, 0.81)***
Offline social capital	−0.28 (-0.40, −0.16)***	−0.10 (-0.27, 0.07)
Online social capital* Offline social capital		−0.01 (-0.02, −0.00)**
Adjusted R^2^	0.3266	0.3515

Notes: Coefficient (95% confidence intervals) are shown. Both models also control for race, age, gender, education, current schooling status, employment, and years in the US. CESD-R = Center for Epidemiologic Studies Depression Scale Revised. DACA = Deferred Action for Childhood Arrivals. Ref = Referent category. Model 6 assesses the relationship between DACA status and online/offline social capital and depressive symptoms after controlling for sociodemographic characteristics. Model 7 examines offline social capital as a potential moderator of the relationship between online social capital and depressive symptoms after controlling for DACA status and sociodemographic characteristics.

*p < 0.05, **p < 0.01, ***p < 0.001.

## 4 Discussion

This study found that those with DACA have lower levels of depressive symptoms compared to those without DACA. It extends previous literature by examining the role of social capital, finding that online social capital may partially mediate the relationship between DACA status and depressive symptoms. That is, lower levels of online social capital among DACA-recipients may explain the relationship between DACA and depressive symptoms. While these findings are in line with our hypotheses that online social capital may be a marker of immigrant-related stress, it runs counter to the broader social capital literature, which often views social capital as a resource (Ehsan and De Silva, 2015).

Moreover, our study finds that the positive association between online social capital and depressive symptoms becomes less pronounced as offline social capital increases. For those without DACA who appear to rely more on online interaction, in-person community may play an especially important role in protecting mental health. DACA status confers a number of benefits, including the ability to work, go to school, and use health services without the fear of deportation (Sudhinaraset et al., 2017). These benefits allow undocumented young adults to participate in formal institutions in the physical world, connecting young people to expanded social networks. This study also found that those without DACA are more likely to have higher online social capital. This is in line with research that suggests that while undocumented immigrants may have risk mitigation strategies in the physical world, they are less likely to possess the same level of caution online (Guberek et al., 2018). Therefore, this suggests that those without DACA may replace their physical space interactions with intimate engagements online.

To our knowledge, no studies exist on online social capital among undocumented young adults. This is a particularly important population to understand these dynamics given the unique stressors faced related to surveillance, privacy, and harassment by anti-immigrant groups (Guberek et al., 2018). One systematic review of social networking sites and depression and anxiety found that, overall, the technology was related to less loneliness and greater self-esteem and life satisfaction (Seabrook et al., 2016). Other studies have also demonstrated how the use of online communication platforms, like WhatsApp, can increase online social capital resources and positively influence psychological well-being (Bano et al., 2019, Guo et al., 2014). For immigrants who use social networking sites, more heterogeneous strong ties (i.e. bridging social capital) have been reported, potentially leading to greater integration and employment opportunities (Binder and Sutcliffe, 2014, Damian and Van Ingen, 2014). Future studies should attempt to differentiate between different online platforms (e.g., social networking sites versus online forums) and the types of online communication taking place as some studies suggest a nuanced relationship between online social relationships and mental health outcomes. For example, Baek et al. (2013) showed that the use of social networking sites like Twitter and Facebook can either improve or harm one’s psychological wellbeing depending on the level of reciprocity and authenticity of the relationship between users (Baek et al. (2013). Lastly, this study also found that offline social capital moderated the relationship between online social capital and depressive symptoms. This highlights the power of offline social capital and the need to continue to foster community engagement in physical spaces, particularly for the most vulnerable.

There are several limitations to this study, including those related to the internet-based data collection method. Our age distribution of DACA-recipients is similar to national estimates using USCIS data from 2017, with slightly more DACA women with higher educational attainment (i.e. USCIS data report 53% of DACA recipients were female and only 13–14% of DACA recipients having greater than a high school degree/GED) (USCIS, 2017). Our sample demographics likely reflect our online recruitment methods and therefore may not be representative of DACA or undocumented populations at large (Dewitt et al., 2018). We used logic edits based on immigrant-related immigration-history questions and DACA status that resulted in an attrition of nearly half the sample. This attrition is in line with other internet-based samples of undocumented immigrants with DACA status (Gonzales et al., 2014, Wong and Valdivia, 2014, Wong et al., 2015). In online survey studies of hard-to-reach populations, there are several tools that can help to detect possible spam respondents, but it remains challenging to comprehensively validate participant reported identity demographics, such as sexual orientation, gender, and immigration status, that do not carry any specific defining criteria, as opposed to highly specific categorizations like DACA status (Dewitt et al., 2018). It is also plausible that conducting surveys in-person could have resulted in a sample with lower levels of online social capital. Relatedly, the online recruitment methods used may have resulted in collider bias in associations between online social capital and depressive symptoms. That is, a person with both higher levels of online social capital and depressive symptoms may cause a person to be online more and, thus, more likely to be selected into the study. One strength of the data is that it directly asks participants about their documentation status as opposed to relying on assumptions and algorithms.

Other limitations of the study include offering the survey in English-only, which may have biased our sample towards more acculturated or educated youth. This study takes place in California, a state that is relatively more inclusive of immigrants compared to other US states. Differences in online and offline social capital and the influence on health may be even more pronounced in less welcoming states. The cross-sectional nature of the survey also does not lend itself to establishing causality. Longitudinal data is particularly important for understanding mediation across legal status and depression, and reverse causation may be an issue. For example, while we hypothesize that spending time online may lead to depression, it is also plausible that depression may lead individuals to disassociate from in-person communities and spend more time online. Lastly, an important limitation is that we do not have sufficient sample to analyze sub-ethnicities among Asians and Pacific Islanders and Latinos. We recognize that both are extremely heterogeneous, with rich histories and unique cultural practices and identities.

Despite these limitations, this study points to several recommendations and future areas of research. First, there is a need for public health practitioners to engage actors not traditionally included in health interventions, such as Facebook, Instagram, and other social media companies. Other researchers, for example, have called for companies such as Facebook to provide a virtual sanctuary space for undocumented immigrants (Guberek et al., 2018). Additionally, social networking sites and users alike should take extra precautions to verify the validity of shared information affecting undocumented immigrants. For instance, dissemination of misinformation about ICE raids or exclusion policies, such as public charge, can cause unnecessary fear, anxiety, and “chilling effects” amongst undocumented and immigrant communities (Perreira et al., 2018). This study also points to the power of offline communities and the need to continue investing in CBOs, training counselors, and offering educational opportunities in schools to engage young adult undocumented immigrants. Given the high frequency of un-moderated virtual communities and peer-to-peer online engagement, future research is needed to examine how to effectively combine in-person community-based support with trusted, online communities.

## Funding

This study was funded by the 10.13039/100010336Hellman Foundation (Fund 7082132). The funding source had no role in the design and conduct of the study; collection, management, analysis, and interpretation of the data; preparation, review, or approval of the manuscript; and decision to submit the manuscript for publication.

## CRediT authorship contribution statement

**May Sudhinaraset:** Conceptualization, Methodology, Data curation, Investigation, Writing – original draft. **Amanda Landrian:** Methodology, Investigation, Writing – original draft. **Hye Young Choi:** Data curation, Methodology, Investigation, Writing – original draft. **Irving Ling:** Conceptualization, Methodology, Investigation, Writing – original draft.

## Declaration of Competing Interest

The authors declare that they have no known competing financial interests or personal relationships that could have appeared to influence the work reported in this paper.

## References

[b0005] Annual Survey|California Emerging Technology Fund [Internet]. [cited 2017 Apr 20]. Available from: http://www.cetfund.org/progress/annualsurvey.

[b0010] Baek, Y.M., Bae, Y., Jang, H., 2013. Social and Parasocial Relationships on Social Network Sites and Their Differential Relationships with Users’ Psychological Well-Being | Cyberpsychology, Behavior, and Social Networking. Cyberpsychology Behav Soc Netw [Internet]. 2013 [cited 2021 Jul 28];16. Available from: https://www.liebertpub.com/doi/full/10.1089/cyber.2012.0510?casa_token=PDbNrcfpZ2MAAAAA%3AfJTN-R1VjrrGI0fudrf8pR6v1HWfRuNpEZDOWDiG3ifisbehEdqsct2DCp-W_dSZAZ7dVlBeHHev. 10.1089/cyber.2012.0510 23697533

[b0015] Bano S., Cisheng W., Khan A.N., Khan N.A. (2019). WhatsApp use and student’s psychological well-being: Role of social capital and social integration. Child Youth Serv. Rev..

[b0020] Baron R., Kenny D. (1986). The moderator-mediator variable distinction in social psychological research: conceptual, strategic, and statistical considerations. J. Pers. Soc. Psychol..

[b0025] Binder J.F., Sutcliffe A.G. (2014). The Best of Both Worlds? Online Ties and the Alternating Use of Social Network Sites in the Context of Migration. Societies.

[b0030] Björgvinsson T., Kertz S.J., Bigda-Peyton J.S., McCoy K.L., Aderka I.M. (2013). Psychometric properties of the CES-D-10 in a psychiatric sample. Assessment.

[b0035] Damian E., Van Ingen E. (2014). Social Network Site Usage and Personal Relations of Migrants. Societies..

[b0040] Davila E.P., Kolodziejczyk J.K., Norman G.J., Calfas K., Huang J.S., Rock C.L. (2014). Relationships between depression, gender, and unhealthy weight loss practices among overweight or obese college students. Eat. Behav..

[b0045] Dewitt J., Capistrant B., Kohli N., Rosser B.R.S., Mitteldorf D., Merengwa E. (2018). Addressing Participant Validity in a Small Internet Health Survey (The Restore Study): Protocol and Recommendations for Survey Response Validation. JMIR Res Protoc..

[b0050] Ehsan A., De Silva M. (2015). Social capital and common mental disorder: a systematic review | Journal of Epidemiology & Community Health. J. Epidemiol. Community Health.

[b0055] Facebook users worldwide 2019 [Internet]. Statista. [cited 2019 Aug 9]. Available from: https://www.statista.com/statistics/264810/number-of-monthly-active-facebook-users-worldwide/.

[b0060] Garcini L.M., Peña J.M., Galvan T., Fagundes C.P., Malcarne V., Klonoff E.A. (2017). Mental Disorders Among Undocumented Mexican Immigrants in High-Risk Neighborhoods: Prevalence, Comorbidity, and Vulnerabilities. J. Consult. Clin. Psychol..

[b0065] Gonzales, R.G., Bautista-Chavez, A., 2014. Two Years and Counting: Assessing the Growing Power of DACA [Internet]. American Immigration Council; 2014. Available from: http://www.immigrationpolicy.org/sites/default/files/docs/two_years_and_counting_assessing_the_growing_power_of_daca_final.pdf.

[b0070] Guberek, T., McDonald, A., Simioni, S., Mhaidli, A.H., Toyama, K., Schaub, F., 2018. Keeping a Low Profile?: Technology, Risk and Privacy among Undocumented Immigrants. Proc 2018 CHI Conf Hum Factors Comput Syst - CHI 18 [Internet]. Montreal QC, Canada: ACM Press; 2018 [cited 2019 Aug 9]. p. 1–15. Available from: http://dl.acm.org/citation.cfm?doid=3173574.3173688.

[b0075] Gonzales R.G., Suárez-Orozco C., Dedios-Sanguineti M.C. (2013). No Place to Belong: Contextualizing Concepts of Mental Health Among Undocumented Immigrant Youth in the United States. Am. Behav. Sci..

[b0080] Gonzales R.G., Terriquez V., Ruszczyk S.P. (2014). Becoming DACAmented: Assessing the Short-Term Benefits of Deferred Action for Childhood Arrivals (DACA). Am. Behav. Sci..

[b0085] Guo, Y, Li, Y., Ito, N., 2014. Exploring the Predicted Effect of Social Networking Site Use on Perceived Social Capital and Psychological Well-Being of Chinese International Students in Japan | Cyberpsychology, Behavior, and Social Networking. Cyberpsychology Behav Soc Netw [Internet]. 2014 [cited 2021 Jul 28];17. Available from: https://www.liebertpub.com/doi/full/10.1089/cyber.2012.0537?casa_token=XSmR8m8uhXsAAAAA%3AROkWWIi3KbsI1joYXPr37IyLqhyB79Occ-4zTOAP0v5k2Yfr6sM4g2LSarkHdAEvXvgWnNOWrGqm&. 10.1089/cyber.2012.0537 23971431

[b0090] Ince J., Rojas F., Davis C.A. (2017). The social media response to Black Lives Matter: how Twitter users interact with Black Lives Matter through hashtag use. Ethn Racial Stud..

[b0095] Manjoo F., 2016. For Millions of Immigrants, a Common Language: WhatsApp. N Y Times [Internet]. 2016 Dec 21 [cited 2017 Jun 30]; Available from: https://www.nytimes.com/2016/12/21/technology/for-millions-of-immigrants-a-common-language-whatsapp.html.

[b0100] Nie N.H., Erbring L. (2002). Internet and society: A preliminary report. IT Soc..

[b0105] Nie N.H., Hillygus D.S. (2002). The Impact of Internet Use on Sociability: Time Diary Findings. IT Soc..

[b0110] Patler C., Laster Pirtle W. (2018). From undocumented to lawfully present: Do changes to legal status impact psychological wellbeing among latino immigrant young adults?. Soc. Sci. Med..

[b0115] Pearlin L.I., Menaghan E.G., Lieberman M.A., Mullan J.T. (1981). The Stress Process. J. Health Soc. Behav..

[b0120] Perreira K.M., Yoshikawa H., Oberlander J. (2018). A New Threat to Immigrants’ Health — The Public-Charge Rule. N. Engl. J. Med..

[b0125] Potochnick S.R., Perreira K.M. (2010). Depression and Anxiety among First-Generation Immigrant Latino Youth: Key Correlates and Implications for Future Research. J. Nerv. Ment. Dis..

[b0130] Ro, A., Van Hook, J., 2021. Comparing the Effectiveness of Assignment Strategies for Estimating Likely Undocumented Status in Secondary Data Sources for Latino and Asian Immigrants | SpringerLink. Popul Res Policy Rev [Internet]. 2021 [cited 2021 Jul 28]; Available from: https://link.springer.com/article/10.1007/s11113-021-09658-3.

[b0135] Ro A., Nakphong M.K., Choi H.Y., Nguyen A., Sudhinaraset M. (2021). The association between social ties and depression among Asian and Pacific Islander undocumented young adults. BMC Public Health..

[b0140] Sacchetti, M., 2021. U.S. judge blocks new applicants to program that protects undocumented ‘dreamers’ who arrived as children. Wash Post [Internet]. 2021 [cited 2021 Jul 19]; Available from: https://www.washingtonpost.com/immigration/daca-court-decision/2021/07/16/6c9a35be-e677-11eb-a41e-c8442c213fa8_story.html.

[b0145] Seabrook, E.M., Kern, M.L., Rickard, N.S., 2016. Social Networking Sites, Depression, and Anxiety: A Systematic Review. JMIR Ment Health [Internet]. 2016;3. Available from: http://www.ncbi.nlm.nih.gov/pmc/articles/PMC5143470/. 10.2196/mental.5842 PMC5143470 27881357

[b0150] Spottswood E.L., Wohn D.Y. (2020). Online social capital: recent trends in research. Curr. Opin. Psychol..

[b0155] Stein G.L., Kiang L., Supple A.J., Gonzalez L.M. (2014). Ethnic identity as a protective factor in the lives of Asian American adolescents. Asian Am. J. Psychol. US: Educ. Publ. Foundation.

[b0160] Sudhinaraset, M., To, T.M., Ling, I., Melo, J., Chavarin, J., 2017. The Influence of Deferred Action for Childhood Arrivals on Undocumented Asian and Pacific Islander Young Adults: Through a Social Determinants of Health Lens. J. Adolesc. Health [Internet]. 2017 [cited 2017 Mar 28];0. Available from: http://www.jahonline.org/article/S1054-139X(17)30051-4/abstract. 10.1016/j.jadohealth.2017.01.008 28359735

[b0165] Sudhinaraset M., To T.M., Ling I., Melo J., Chavarin J. (2017). The Influence of Deferred Action for Childhood Arrivals on Undocumented Asian and Pacific Islander Young Adults: Through a Social Determinants of Health Lens. J. Adolesc. Health Off Publ. Soc. Adolesc. Med..

[b0170] The Role of Social Media in the Arab Uprisings | Pew Research Center [Internet] cited 2019 Aug 9 Available from: https://www.journalism.org/2012/11/28/role-social-media-arab-uprisings/, 2012.

[b0175] Turner, R.J., 2009. Understanding Health Disparities: The Promise of the Stress Process Model. In: Avison WR, Aneshensel CS, Schieman S, Wheaton B, editors. Adv Conceptualization Stress Process [Internet]. Springer New York; 2009 [cited 2017 Jun 17]. p. 3–21. Available from: http://link.springer.com/chapter/10.1007/978-1-4419-1021-9_1.

[b0180] USCIS. DACA Recipients [Internet]. US Citizenship and Immigration Services: USCIS; 2017. Available from: https://www.uscis.gov/sites/default/files/document/data/daca_population_data.pdf.

[b0185] Venkataramani A.S., Shah S.J., O'Brien R., Kawachi I., Tsai A.C. (2017). Health consequences of the US Deferred Action for Childhood Arrivals (DACA) immigration programme: a quasi-experimental study. Lancet Public Health.

[b0190] Williams D. (2006). On and Off the ’Net: Scales for Social Capital in an Online Era. J. Comput. Mediat. Commun..

[b0195] Wolfsfeld, G., Segev, E., Sheafer, T., 2013. Social Media and the Arab Spring: Politics Comes First. Int J Press [Internet]. 2013 [cited 2019 Aug 9]; Available from: https://journals.sagepub.com/doi/full/10.1177/1940161212471716.

[b0200] Wong T, Valdivia C. In Their Own Words: A Nationwide Survey of Undocumented Millenials [Internet]. United We Dream Network; 2014. Available from: http://unitedwedream.org/words-nationwide-survey-undocumented-millennials/.

[b0205] Wong, T.K., Richter, K.K., Rodriguez, I., Thursday, P.E.W., 9 J, 2015. Results from a Nationwide Survey of DACA Recipients Illustrate the Program’s Impact [Internet]. Available from: https://www.americanprogress.org/issues/immigration/news/2015/07/09/117054/results-from-a-nationwide-survey-of-daca-recipients-illustrate-the-programs-impact/.

[b0210] Zong, J., Batalova, J., Burrows, M., 2019. Frequently Requested Statistics on Immigrants and Immigration in the United States [Internet]. migrationpolicy.org. 2019 [cited 2019 Jun 11]. Available from: https://www.migrationpolicy.org/article/frequently-requested-statistics-immigrants-and-immigration-united-states.

