# Supplementary material for: Redefining communities: The association between deferred action, online and offline social capital and depressive symptoms among undocumented young adults
Source: Prev Med Rep. 2021 Sep 20;24:101563. doi: 10.1016/j.pmedr.2021.101563 (PMC8683852; doi:10.1016/j.pmedr.2021.101563)
Supplement: Supplementary data 1 [file mmc1.docx]

**Appendix A: Internet Social Capital Scale (ISCS),** based on Williams, 2006

| 1. There are several people online* I trust to help solve my problems. |
| --- |
| 1. There is someone online* I can turn to for advice about making very important decisions. |
| 1. There is no one online* that I feel comfortable talking to about intimate personal problems. |
| 1. When I feel lonely, there are several people online* I can talk to. |
| 1. The people I interact with online* would help me fight an injustice. |
| 1. Interacting with people online* makes me interested in things that happen outside of my town. |
| 1. Interacting with people online* makes me interested in what people unlike me are thinking. |
| 1. Interacting with people online* makes me feel like part of a larger community. |
| 1. Interacting with people online* makes me feel connected to the bigger picture.   *The same set of questions were asked for “offline” networks. Instead of “online” we used “offline.” |
